# Serum lipase activity and concentration during intravenous infusions of GLP‐1 and PYY_3‐36_ and after ad libitum meal ingestion in overweight men

**DOI:** 10.14814/phy2.12980

**Published:** 2016-09-26

**Authors:** Julie B. Schmidt, Anders Sjödin, Lene S. Stevner, Christian Ritz, Natasha B. Michaelsen, Anne B. Thomsen, Jens J. Holst, Arne Astrup

**Affiliations:** ^1^ Department of NutritionExercise and SportsFaculty of ScienceUniversity of CopenhagenCopenhagenDenmark; ^2^ Research BioanalysisNovo Nordisk A/SMåløvDenmark; ^3^ Medical & ScienceGLP‐1 & ObesityNovo Nordisk A/SBagsvaerdDenmark; ^4^ NNF Center for Basic Metabolic Research and Department of Biomedical SciencesFaculty of Health SciencesUniversity of CopenhagenCopenhagenDenmark

**Keywords:** GLP‐1, PYY_3‐36_ and serum lipase

## Abstract

To examine the effect on serum lipase activity and protein concentration of intravenous infusions of glucagon‐like peptide‐1 (GLP‐1) and peptide YY (PYY_3‐36_) and of an ad libitum meal in healthy overweight men. Twenty‐five healthy, male subjects participated in this randomized, double‐blinded, placebo‐controlled 4‐arm crossover study (Body Mass Index (BMI): 29 ± 3 kg/m^2^, age: 33 ± 9 years). On separate days, the subjects received a 150‐min intravenous infusion of either (1) 0.8 pmol/kg/min PYY_3‐36_, (2) 1.0 pmol/kg/min GLP‐1, (3) 1 + 2, or (4) placebo. Samples were collected throughout the infusion and after intake of an ad libitum meal for measurement of serum lipase. Serum lipase levels measured by enzyme‐linked immunosorbent assay (ELISA) following mono‐infusions of GLP‐1 and PYY_3‐36_ were comparable to serum lipase levels following placebo (*P *=* *0.054 and *P *=* *0.873, respectively). Following the co‐infusion of GLP‐1 and PYY_3‐36_, serum lipase levels measured by ELISA decreased over time compared to placebo (*P *=* *0.012). However, the between‐group difference was not consistent when each time point was analyzed separately. On the placebo day, serum lipase levels measured by ELISA after an ad libitum meal rose slightly compared to the preprandial values (*P* = 0.003). There was strong correlation between serum lipase levels measured by ELISA and LIPC Lipase colorimetric assay (COBAS) (0.94 < *r*; <0.0001). Infusions of GLP‐1 and PYY_3‐36_, separately or in combination, did not increase serum lipase. However, a small increase in serum lipase may occur in response to a meal.

## Introduction

Changes in the secretion of the gastro‐intestinal hormones, glucagon‐like peptide‐1 (GLP‐1) and peptide YY (PYY), are believed to play an important role for weight loss after Roux‐en‐Y gastric bypass (RYGB) (le Roux et al. 2007; Jacobsen et al. 2012). In addition to its role as an important regulator of appetite, GLP‐1 is also involved in the postoperative remission of type 2 diabetes mellitus (T2D) (le Roux et al. 2007; Jorgensen et al. 2013).

Agonists of the GLP‐1 receptor (GLP‐1R), including exenatide (Byetta^®^) and liraglutide (Victoza^®^) have been approved for the treatment of T2D and these drugs effectively reduce blood glucose, body weight, and systolic blood pressure. The mechanism by which GLP‐1R agonists reduce blood glucose involves potentiation of glucose‐induced insulin secretion from the pancreatic *β*‐cells (Hare et al. 2009). It has also been shown that the production of glucose from the liver is reduced during treatment with liraglutide via an inhibitory effect on glucagon secretion (Hare et al. 2009). Thus, the effect of GLP‐1R agonists on the endocrine part of the pancreas is well documented, however, whether there is any effect of these agonists on the exocrine part of the pancreas is not clear. There are data indicating that GLP‐1Rs are only present on the pancreatic *β*‐cells, while other data indicate that the acinar cells of the exocrine pancreas also contain GLP‐1Rs at a lower density (Pyke et al. 2014; Waser et al. 2015).

The enzyme pancreatic lipase is released from the acinar cells of the exocrine part of the pancreas to the duodenum during a meal and acts to hydrolyze triglycerides. In the clinic, this enzyme is used as a marker of pancreatic damage. An increase in serum pancreatic lipase at approximately 10–12 U/L has been reported after 2–4 weeks of treatment in clinical studies with several GLP‐1R agonists (Bastyr et al. 2009; Steinberg et al. 2012). Although this increase is within the normal range (13–60 U/L) (DeVries et al. 2012), the mechanism needs further investigation.

Whether the release of lipase to the gut during a meal is also accompanied by an increase in serum lipase, as a part of a normal postprandial response, is unclear. Thus, it would be of interest to measure serum lipase in the pre‐ and postprandial phases of a meal. Acute changes in the levels of serum lipase in patients who are treated with native GLP‐1 or PYY_3‐36_ have not been reported either, and such a study would be relevant in order to elucidate whether these peptides have an immediate and direct effect on the exocrine part of the pancreas.

Previously, we investigated the effect of intravenous infusion of GLP‐1 and PYY_3‐36_, separately and in combination, on energy intake, energy expenditure, and appetite sensations in healthy overweight men (Schmidt et al. 2014). In this post hoc substudy, we included additional analyses to elucidate whether infusion of GLP‐1 and PYY_3‐36_ as well as an ad libitum meal increased serum lipase. Furthermore, we compared the standard assay used in most clinical trials for assessment of lipase activity, the LIPC Lipase colorimetric assay (COBAS) with a dedicated enzyme‐linked immunosorbent assay (ELISA) assay for the human lipase protein.

## Methods

### Study population

Subjects included 25 moderately overweight (BMI ≥ 25), healthy Caucasian men, aged 18–50 years (Schmidt et al. 2014). The study was approved by the Municipal Ethical Committee of Copenhagen/Scientific Ethics Committee of the Capital Regions of Denmark (journal no. H‐1‐2009‐083 and H‐3‐2013‐091) and carried out in accordance with the Helsinki‐II Declaration/Declaration of Helsinki and by the Danish Data Protection Agency (journal no. 2007‐54‐0296). The study followed the guidelines of Good Clinical Practice (GCP) and was registered at Clinical Trials (ID# NCT00940134).

### Study design

**Figure 1:**
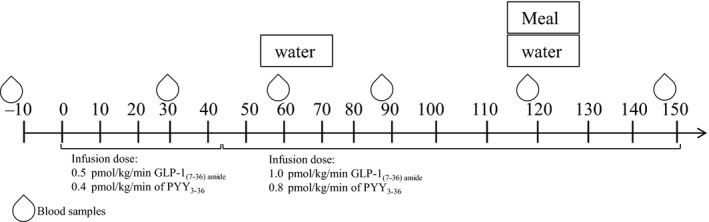
Overview of a test day. Infusion of GLP‐1, PYY_3‐36_, GLP‐1 + PYY_3‐36_, or placebo was initiated at time 0 and continued for 150 min while the ad libitum meal was served at time 120. GLP‐1, glucagon‐like peptide‐1; PYY, peptide YY.

As described previously (Schmidt et al. 2014), the study was a randomized, double‐blinded, 4‐arm crossover design. Subjects attended the Department of Nutrition, Exercise and Sports on four separate test days and received a 150‐min intravenous infusion of either (1) GLP‐1, (2) PYY_3‐36_, (3) GLP‐1 + PYY_3‐36,_ or (4) placebo. Doses of 0.5 pmol/kg/min of GLP‐1_7‐36_ amide and 0.4 pmol/kg/min of PYY_3‐36_ for the first 45 min, and 1.0 pmol/kg/min GLP‐1_7‐36_ amide and 0.8 pmol/kg/min of PYY_3‐36_ for the remaining 105 min were chosen (Fig. 1). Synthetic GLP‐1_7‐36_ amide and PYY_3‐36_ from polypeptides (Wolfenbüttel, Germany) with a purity of >97% were used. Purity and structure were estimated by high‐performance liquid chromatography (HPLC) analysis, mass spectrometry, and sequence analysis. PYY_3‐36_ and GLP‐1_7‐36_ amide alone or in combination, were dissolved in saline with 2% human albumin in order to limit adhesion to infusion material and the solutions were distributed in glass tubes. The solutions and placebo (saline and albumin only) were prepared according to Good Manufacturing Practice (GMP) and tested for sterility and pyrogenic content at the Capital Region Pharmacy, Herlev. Treatments were given in a random order, and both the subjects and the researchers involved with the subjects were blinded to the treatments.

On each test day (Visit 1–4) (Fig. 1), the subject arrived at 7:30 am after an overnight fast. To limit fluctuations in glycogen stores and energy balance, subjects received a standardized dinner, and were instructed to refrain from alcohol and strenuous physical activities on the day prior to the test days. Intravenous catheters were inserted into an antecubital vein in each arm; one for hormone infusion and another for sampling of blood. Infusions began at time 0 and were continued for a total of 150 min. Blood samples were collected every 30 min and the same measurements as those collected at baseline were repeated. After 120 min, an ad libitum meal was served. Thirty minutes after initiation of the meal (150 min after start of infusion), the last blood samples were drawn before the infusion was stopped.

### Biochemical measures

#### ELISA for determination of total lipase concentration

This sandwich ELISA was originally developed by Grandval et al. (2004). In brief, analyses were performed in 96‐well polyvinyl chloride (PVC) microtiter plates (Maxisorp, Nunc). Plates were coated at 4°C overnight with a purified rabbit anti‐human pancreatic lipase polyclonal antibody used as a catcher antibody. Plates were washed and the standards of human pancreatic lipase were prepared at concentrations ranging from 0 to 1–5 μg/L as well as unknown plasma samples were added to the plates. Incubation proceeded at 37°C for 1 h. After a washing step, a biotinylated monoclonal antibody specific for human pancreatic lipase was added to the plates, followed by one hour incubation at 4°C. A solution of ExtrAvidin peroxidase conjugate was added and the plates were incubated at 37°C for 45 min. The plates were washed three times and freshly prepared peroxidase substrate solution was added to each well. The plates were kept at room temperature in the dark for 15 min and the enzyme reaction was stopped by the addition of 4 mol/L H_2_SO_4_ to each well. The optical density was measured at 450–620 nm using a Tecan Sunrise spectrophotometer.

#### LIPC Lipase colorimetric assay

This method is based on the cleavage of a specific chromogenic lipase substrate 1,2‐O‐dilauryl‐rac‐glycero‐3‐glutaric acid‐(6‐methylresorufin) ester emulsified with bile acids. The pancreatic enzyme activity was determined specifically by the combination of bile acid and colipase used in this assay. Virtually, no lipase activity was detected in the absence of colipase. Colipase only activates pancreatic lipase, but not other lipolytic enzymes found in serum. The high amount of cholates ensures that the esterases present in the serum do not react with the chromogenic substrate due to the highly negative surface charge.

### Statistical analysis

Linear mixed models were used for evaluating lipase levels preprandially, one analysis per assay. These models included time‐treatment interactions as well as adjustment for baseline body weight and carry‐over as fixed effects and subject‐by‐period random effects. Concentrations were logarithm‐transformed if needed. Differences in trends over time between intervention groups were evaluated using chi‐square tests. Pairwise comparisons between groups per time points were also made (using model‐based *t*‐tests). Model checking was based on visual inspection of residual and normal probability plots. Postprandially, concentrations were analyzed using analysis of covariance with same adjustments as above. In addition, we adjusted for meal size and concentration recorded prior to the ad libitum meal; these analyses were carried out both for all data (pooling groups) and for placebo data only. Pairwise comparisons between groups were considered. For comparison of the two serum lipase assays, a Pearson correlation was made. Additionally, correlation analyses were made to explore associations between changes in serum lipase levels and meal size. The statistical environment R was used for the analyses (R Core Team, 2012). A significance level of 0.05 was used.

## Results

### The effect of infusion of GLP‐1, PYY_3‐36_ and GLP‐1 + PYY_3‐36_ on lipase levels in serum

**Figure 2:**
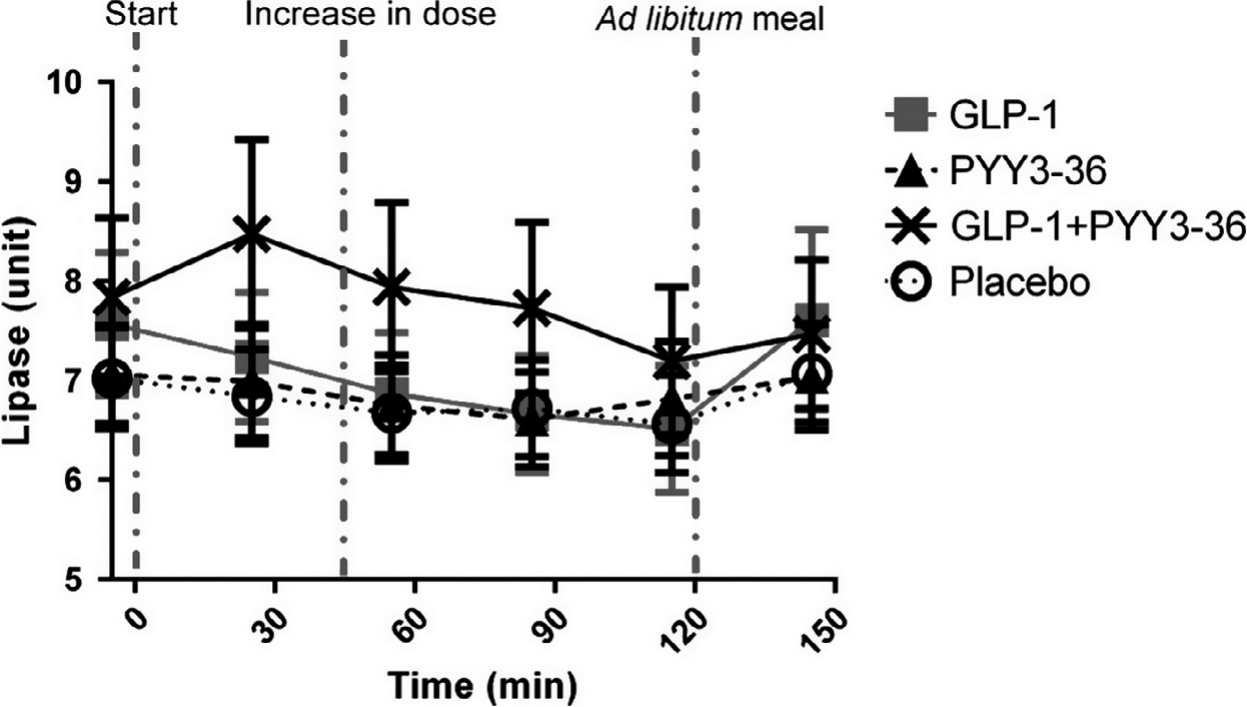
Serum levels of lipase measured by ELISA following infusion of GLP‐1, PYY_3‐36_, GLP‐1 + PYY_3‐36_, and placebo. GLP‐1, glucagon‐like peptide‐1; PYY, peptide YY.

**Figure 3:**
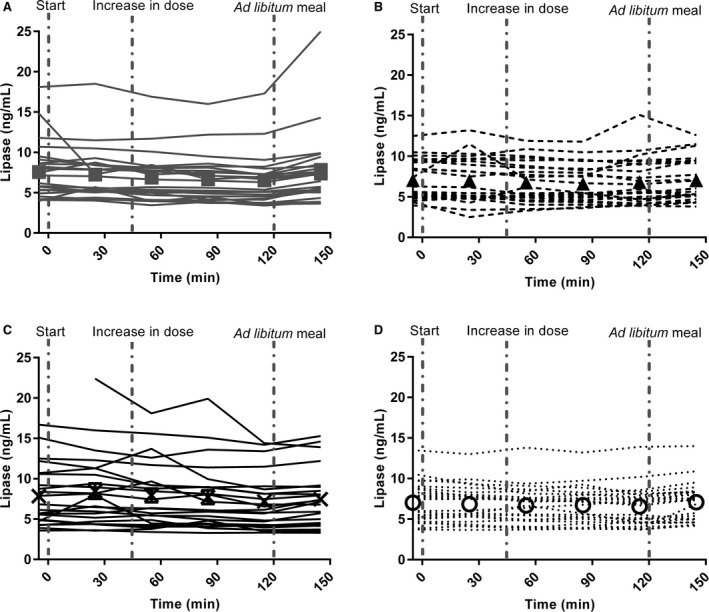
Serum levels of lipase measured by ELISA for the individual subjects following infusion of (A) GLP‐1, (B) PYY_3‐36_, (C) GLP‐1 + PYY_3‐36_, and (D) placebo GLP‐1, glucagon‐like peptide‐1; PYY, peptide YY.

In a model summarizing the trend for all preprandial time points (30–120 min) and taking baseline differences into account, serum lipase levels following mono‐infusions of GLP‐1 and PYY_3‐36_ were comparable to serum lipase levels following placebo (*P *=* *0.054 and *P *=* *0.87, respectively) (Figs. 2 and 3). Following co‐infusion of GLP‐1 and PYY_3‐36,_ serum lipase levels measured by ELISA actually decreased over time compared to placebo (*P *=* *0.012) (Figs. 2 and 3). However, this between‐group difference was no longer present when each time point was analyzed separately.

We performed similar analyses on serum lipase levels measured by COBAS. In agreement with data from the ELISA, we found no between‐group differences when each time point was analyzed separately and following adjustments for multiple testing.

### The effect of a meal on lipase levels in serum

On the placebo day, serum lipase levels measured by ELISA after an ad libitum meal (at 150 min) were higher than preprandial (at 120 min) (*P* = 0.003) (Figs. 2 and 3D). In a model adjusting for the preprandial measurement, the postprandial levels were not different between placebo and the active treatments groups (GLP‐1: *P *=* *0.27, PYY: *P *=* *0.41, co‐infusion: *P *=* *0.60) (Fig. 2). We neither found association between change in serum lipase (postprandial – preprandial) and energy intake during the ad libitum meal in a pooled analysis (*P *=* *0.16) nor when looking at the placebo group separately (*P *=* *0.63). Analyses on serum lipase levels measured by COBAS led to similar conclusions.

### Correlation of lipase levels in serum measured by a standard colorimetric assay versus an immunological ELISA assay

**Figure 4:**
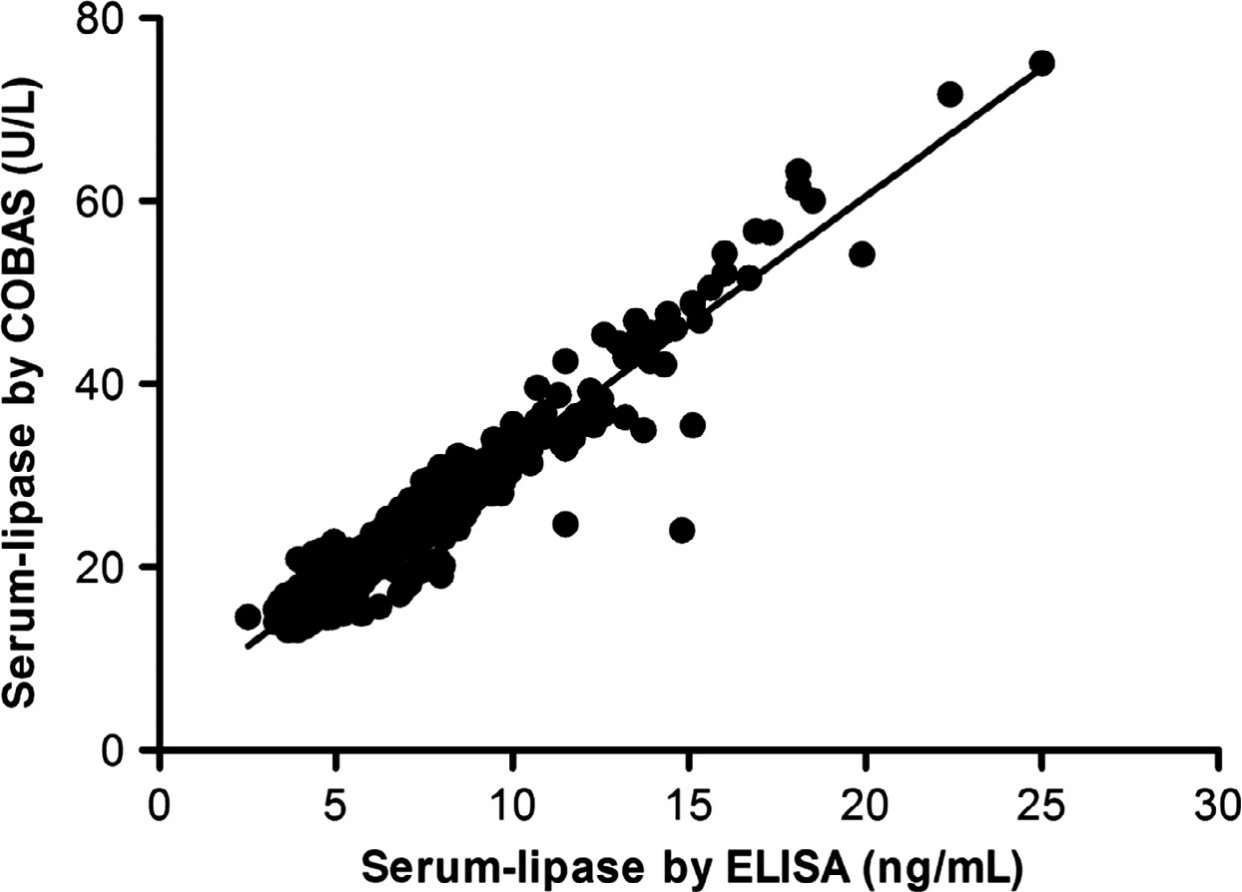
Correlation between levels of lipase in serum measured by COBAS versus ELISA (0.94 < *r* < 0.98; *P *<* *0.0001).

Serum lipase activity was measured by COBAS at fasting, at all preprandial time points, and after an ad libitum meal and these data were compared with data obtained from ELISA. The two methods were highly correlated (0.94 < *r* < 0.98; *P *<* *0.0001) (Fig. 4).

## Discussion

### Effects of the infusions

Our data do not support the initial hypothesis that infusions of GLP‐1 and PYY_3‐36_, separately or in combination, increase levels of lipase in serum. In contrast, the trends over time analysis showed that, if anything, co‐infusion slightly lowered the lipase levels compared to placebo. Similarly, a weak trend toward a decrease in levels of lipase following GLP‐1 infusion compared to placebo was observed.

Evidently, the increase in serum lipase levels observed after weeks of treatment with GLP‐1R agonists in clinical trials could not be confirmed in this “acute” setting and with the levels of GLP‐1 infusion applied in this study. We chose the infusion dose levels based on an attempt to elicit an effect on energy intake while avoiding unacceptable side effects in the form of nausea.

The results from other clinical studies indicate that the serum lipase levels may be higher and more variable in patients with T2D compared with nondiabetic obese patients (DeVries et al. 2012). The reason for this is unknown, but patients with T2D generally have an increased risk of developing pancreatitis as well (Noel et al. 2009). As only healthy subjects were included in this study, we cannot exclude that serum lipase levels would change differently following infusions of GLP‐1 and PYY_3‐36_ in patients with T2D patients, and understanding the mechanism behind the increase in serum lipase that has been reported with Trulicity^®^, Byetta^®^, and Victoza^®^ treatment, therefore, requires further studies conducted on this specific population.

### The effect of a meal

Serum lipase increased slightly following an ad libitum meal on the placebo day. When analyzed together, there was no significant difference in serum lipase between placebo and the different hormone infusions. The increased release of lipase to the intestinal lumen during a meal intake might be expected to be associated with a small increase in plasma levels also, perhaps due to leakage from the duct system or the acinar cells. Whether the effect of a meal on serum lipase levels are modified by infusions of GLP‐1 and PYY_3‐36_ cannot be settled by the present protocol due to differences in meal size resulting from the hormone infusions. Although we found no association between changes in serum lipase and energy intake in pooled analyses, a link between these variables cannot be ruled out entirely. Thus, it is possible that subjects with a naturally high lipase response to a meal are also more sensitive to a stimulatory effect of GLP‐1 or PYY_3‐36_ treatments. Studies with sample size large enough for data to be stratified according to meal responses are therefore warranted.

### COBAS versus ELISA

Most clinical trials have used the COBAS assay for analysis of serum lipase. This assay is relatively specific to pancreatic lipase activity due to the activation by colipase, but the increased activity observed during chronic GLP‐1 RA treatment might actually result from interference on lipase activity by other circulating factors or from hitherto uncharacterized lipolytic activity, rather than an increased release to the blood stream from the pancreas. Therefore, we also analyzed all samples for the pancreatic lipase protein using a sandwich‐type ELISA assay. Upon comparison of the results obtained with the two methods, the correlation between these methods was high, confirming that a tight relationship exists between the plasma pancreatic lipase activity and the actual concentration of the enzyme protein. Based on this comparison, it seems safe to conclude that the activity measured by the COBAS assay is an accurate measure of the presence and therefore the leak/constitutive release of the enzyme from the pancreas.

### Limitations

This study was primarily designed to investigate a potential synergistic effect of GLP‐1 and PYY_3‐36_ on energy intake, and the meal size was therefore not standardized, making it difficult to differentiate between effects of the infusions and effects of the meal when evaluating the postprandial serum lipase levels. Our aim was to include overweight subjects with the assumption that these subjects are at higher risk of metabolic abnormalities and are more eligible for future treatment with a GLP‐1 RA. However, the use of a BMI ≥ 25 as a cut‐off point for this purpose can be criticized, and it is likely that our population consist of a mix of metabolically healthy and unhealthy subjects, who might have different baseline levels and respond differently to exposure of GLP‐1 and PYY_3‐36_. As overweight is not necessarily accompanied by metabolic abnormalities adjusting for body weight, as we did in our analysis, does not overcome this problem.

## Conclusion

Our data do not support that acute infusions of GLP‐1 and PYY_3‐36_, separately or in combination, increase lipase in serum. However, a small increase in serum lipase was observed in response to an ad libitum meal. We found a high correlation between the results of measurements of pancreatic lipase activity and lipase protein in plasma, indicating that the lipase activity is closely related to the presence of the lipase protein.

## Conflict of Interest

None declared.
